# Human UDP-Glucuronosyltransferase 2B4 and 2B7 Are Responsible for Naftopidil Glucuronidation *in Vitro*

**DOI:** 10.3389/fphar.2017.00984

**Published:** 2018-01-11

**Authors:** Xia-Wen Liu, Yi Rong, Xing-Fei Zhang, Jun-Jun Huang, Yi Cai, Bi-Yun Huang, Liu Zhu, Bo Wu, Ning Hou, Cheng-Feng Luo

**Affiliations:** ^1^Key Laboratory of Molecular Clinical Pharmacology and Fifth Affiliated Hospital, Guangzhou Medical University, Guangzhou, China; ^2^State Key Laboratory of Drug Metabolism, Hematological Pharmacology, Beijing Institute of Transfusion Medicine, Beijing, China; ^3^Guangzhou Institute of Cardiovascular Disease, The Second Affiliated Hospital of Guangzhou Medical University, Guangzhou, China

**Keywords:** naftopidil, enantiomer, metabolism, UDP-glucuronosyltransferases, microsomes

## Abstract

Naftopidil (NAF) is widely used for the treatment of benign prostatic hyperplasia and prevention of prostate cancer in elderly men. These patients receive a combination of drugs, which involves high risk for drug–drug interaction. NAF exhibits superior efficacy but must be administered at a much higher dosage than other therapeutic drugs. We previously showed that extensive glucuronidation of NAF enantiomers caused poor bioavailability. However, the metabolic pathway and mechanism of action of NAF enantiomer remain to be elucidated. The present study was performed to identify the human UDP-glucuronosyltransferases (UGTs) responsible for the glucuronidation of NAF enantiomers and to investigate the potential inhibition of UGT activity by NAF. The major metabolic sites examined were liver and kidney, which were compared with intestine. Screening of 12 recombinant UGTs showed that UGT2B7 primarily contributed to the metabolism of both enantiomers. Moreover, enzyme kinetics for R(+)-NAF, UGT2B7 (mean *K*_m_, 21 μM; mean *V*_max_, 1043 pmol/min/mg) showed significantly higher activity than observed for UGT2B4 and UGT1A9. UGT2B4 (mean *K*_m_, 55 μM; mean *V*_max_, 1976 pmol/min/mg) and UGT2B7 (mean *K*_m_, 38 μM; mean *V*_max_, 1331 pmol/min/mg) showed significantly higher catalysis of glucuronidation of S(-)-NAF than UGT1A9. In human liver microsomes, R(+)-NAF and S(-)-NAF also inhibited UGT1A9: mean *K*_i_ values for R(+)-NAF and S(-)-NAF were 10.0 μM and 11.5 μM, respectively. These data indicate that UGT2B7 was the principal enzyme mediating glucuronidation of R(+)-NAF and S(-)-NAF. UGT2B4 plays the key role in the stereoselective metabolism of NAF enantiomers. R(+)-NAF and S(-)-NAF may inhibit UGT1A9. Understanding the metabolism of NAF enantiomers, especially their interactions with metabolic enzymes, will help to elucidate potential drug–drug interactions and to optimize the administration of this medicine.

## Introduction

Naftopidil (NAF) is a specific α_1D/1A_-adrenoceptor blocker frequently used to treat benign prostatic hyperplasia (BPH). Compared with other α_1_-adrenoceptor blockers (i.e., tamsulosin), NAF exhibits superior efficacy (Masumori, 2011). We previously showed that NAF enantiomers can relax the prostate muscle and inhibit prostate growth. S(-)-NAF has been reported to cause stronger inhibition than R(+)-NAF and racemate NAF (Huang et al., 2016). NAF was recently reported to elicit growth-insensitive human prostatic cancer cell lines (Kanda et al., 2008; Yamada et al., 2013). In addition, combination with NAF synergized the efficacy of radiotherapy and docetaxel in treatment of prostate cancer (Ishii et al., 2017; Iwamoto et al., 2017). These previous studies indicate that NAF may have previously unforeseen effects in the treatment of BPH.

NAF exhibited low bioavailability (20% in human and 9% in rat) because of its extensive first-pass metabolism (Himmel, 1994). Thus, the dosage of NAF (25–75 mg/day) is much higher than that of other therapies used to treat BPH (e.g., 0.2 mg/day for tamsulosin, 1–5 mg/day for terazosin, and 5 mg/day for finasteride). At high NAF doses (100 mg/day), animals showed reduced food consumption and body weight gain, apathy, diarrhea, proteinuria, and slightly increased levels of alkaline phosphatase (Himmel, 1994). We therefore sought to elucidate the mechanism underlying first-pass metabolism of NAF.

Previous studies showed that the relevant metabolic pathways in rat and man are qualitatively similar (Himmel, 1994). Three phase I metabolites [(phenyl) hydroxy-NAF (PHN), (naphthyl) hydroxy-NAF (NHN), and *O*-desmethyl-NAF (DMN)] and two phase II metabolites (NAF and NHN glucuronide conjugates) were identified (Niebch et al., 1991; Li et al., 2006). Phase I metabolism of NAF is mediated by cytochrome P450 enzymes (CYP) 2C9 and CYP2C19 (Zhu et al., 2014). Our previous studies indicated the stereoselective pharmacokinetic profiles of NAF enantiomers. However, total excretion ratios of both NAF enantiomers were extremely low (approximately 5% of doses administered; Liu et al., 2012, 2015). We compared the amounts of the major metabolites mentioned above in rat plasma. Results showed that levels of glucuronides of R(+)-NAF, S(-)-NAF [R(+)-NAF-G, and S(-)-NAF-G] were six- and threefold higher than those of R(+)-NAF and S(-)-NAF. Levels of R(+)-NAF-G and S(-)-NAF-G were also much higher than those of other phase I and phase II metabolites (Liu et al., 2017). We inferred that the major metabolic pathway of NAF involved extensive glucuronidation. Interestingly, UDP-glucuronosyltransferases (UGTs) played a larger role than CYPs in first-pass metabolism of NAF *in vivo*. However, the UGTs involved in metabolism of NAF remain to be identified.

Glucuronidation reactions are catalyzed by UGTs and are responsible for the inactivation and elimination of many compounds, including drugs from all therapeutic classes, dietary chemicals, and endogenous compounds (e.g., bilirubin, bile acids, hydroxysteroids; Evans and Relling, 1999; Knights et al., 2013). Extensively glucuronidated compounds are more likely to be affected by UGT enzyme inducers or inhibitors (Lin and Wong, 2002). As a drug used to treat elderly patients, NAF may be applied in combination with various drugs eliminated by UGTs, such as drugs used to treat cardiovascular disease and hypertension (Wienen et al., 2000; Jones et al., 2010; Takekuma et al., 2012) and non-steroidal anti-inflammatory drugs (Joo et al., 2015). Patients who receive NAF face high risk for UGT-mediated drug–drug interactions and drug–endobiotic interactions.

Therefore, the present study aimed to identify human UGTs responsible for the glucuronidation of NAF enantiomers. Additional experiments were performed to investigate glucuronidation of NAF enantiomers across species and in various types of tissue microsomes. The ability of NAF to inhibit UGT activity was also evaluated. Increased knowledge on the glucuronidation of NAF enantiomers will be beneficial in understanding of the efficiency and toxicity of this drug and preventing potential drug–drug interactions and drug–endobiotic interactions.

## Materials and Methods

### Materials

**FIGURE 1:**
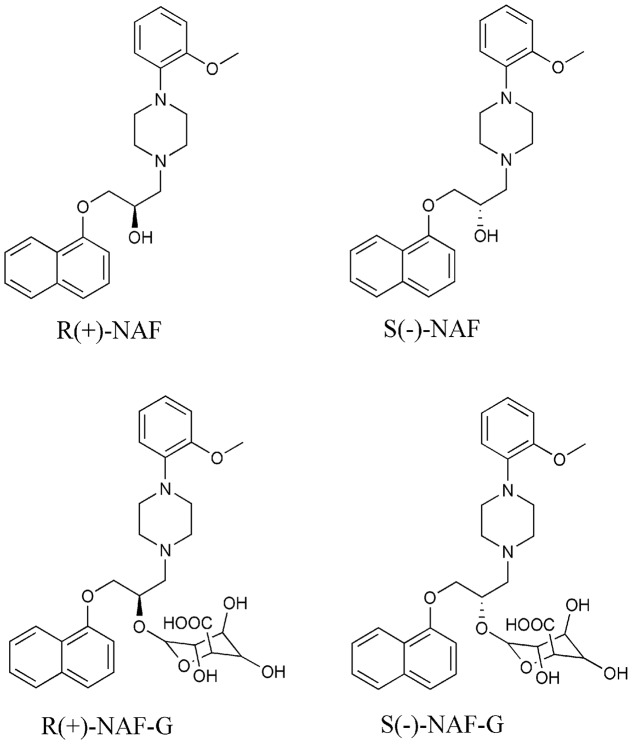
Structures of R(+)-NAF, S(–)-NAF, R(+)-NAF-G, and S(–)-NAF-G.

R(+)-NAF (>99.5% ee) and S(-)-NAF (>99.5% ee) (**Figure 1**) were synthesized according to previous methods (Shivani et al., 2007). The internal standard (IS) Z10 (CN201110148322.7) was obtained from our laboratory. HPLC-grade methanol, acetonitrile, and acetic acid were obtained from Merck (Darmstadt, Germany). Uridine diphosphate glucuronic acid (UDPGA), alamethicin, Tris, magnesium chloride, methylum-belliferone (MU), 4-methylumbelliferone (4-MU), 4-methylum-belliferyl-β-D-glucuronide hydrate (4-MU-G), propofol, propofol glucuronide, zidovudine, zidovudine glucuronide, fluconazole, and niflumic acid, trifluoperazine dihydrochloride (TFP), and TFP-glucuronide (TFP-G) were purchased from Sigma–Aldrich (St. Louis, MO, United States). Recombinant human UGTs (UGT1A1, 1A3, 1A4, 1A6, 1A7, 1A8, 1A9, 1A10, 2B4, 2B7, 2B15, and 2B17) and the commercially available microsomes from human liver (HLMs), intestine (HIMs), and kidney (HKMs), as well as those from rat liver (RLMs), intestine (RIMs), and kidney (RKMs), were purchased from BD Gentest (Woburn, MA, United States). Protein contents of the microsomes were used according to the manufacturers’ instructions. Ultrapure water was used in all experiments (Millipore, Billerica, MA, United States).

### Quantification of R(+)-NAF-G and S(-)-NAF-G

The glucuronidations of NAF enantiomers were quantified as previously described (Liu et al., 2017). Briefly, R(+)-NAF-G and S(-)-NAF-G were biosynthesized using HLMs. The reaction was terminated after incubation for 4 h, when the NAF enantiomer was not detectable. The crude solution was used as stock solutions of R(+)-NAF-G and S(-)-NAF-G. Their final concentrations were determined based on the NAF enantiomer standard curve and the conversion factor of relevant extinction coefficients.

NAF enantiomer glucuronides were detected by rapid resolution liquid chromatography tandem mass spectrometry (RRLC-MS/MS) with multiple reaction monitoring. The instrument parameters for the LC were as follows: system, Agilent 1290; Agilent Eclipse Plus C18 (2.1 mm × 50 mm, 1.8 μm, Santa Clara, CA, United States); mobile phase, methanol: 0.1% formic acid in water = 45:55; flow rate, 0.4 mL/min; column temperature, 25°C; and injection volume, 2 μL. Conditions of Agilent 6460 triple-quadruple MS were set as follows: capillary voltage, -4.0 kV; drying gas temperature, 350°C; nitrogen flow rate, 10 L/min; and nitrogen pressure, 40 psi. The following mass ion transitions (*m/z*) were used for detection: 569.3/393.1 for R(+)-NAF-G and S(-)-NAF-G, and 393.1/190.1 for R(+)-NAF and S(-)-NAF; and 427.0/235.0 for Z10.

### Glucuronidation Assay of NAF Enantiomers

All glucuronidation assays were conducted in an incubation volume of 200 μL. Incubations were performed at 37°C in 50 mM Tris–HCl buffer (pH 7.5) with 10 mM magnesium chloride, 1 mg protein/mL of enzyme (microsomes and recombinant human UGTs), alamethicin (100 μg of alamethicin/mg microsomal protein), and NAF enantiomers (2.5–80 μM). The reaction was initiated by the addition of UDPGA (5 mM) and incubated at 37°C in a shaking water bath for 45 min. The reaction was terminated by the addition of 200 μL of ice-cold acetonitrile containing the IS (1 μM) and then centrifuged at 5000 ×*g* for 10 min at 4°C. The supernatant fraction was injected into the RRLC-MS/MS apparatus. Protein concentration (0.5–2 mg/mL) and incubation time (15–180 min) were optimized in preliminary studies to be within initial linear rate conditions. Preliminary experiments also showed that the samples containing NAF enantiomers, the glucuronidations, and Z10 in the solvent were stable for at least 4 h at 37°C.

### UGT Reaction Screening of NAF Enantiomers

Twelve commercially available recombinant human UGTs (UGT1A1, 1A3, 1A4, 1A6, 1A7, 1A8, 1A9, 1A10, 2B4, 2B7, 2B15, and 2B17) were screened with 10 μM R(+)-NAF and S(-)-NAF by incubation for 45 min. The incubation condition was as described in the Section “Glucuronidation Assay of NAF Enantiomers,” and then the samples were analyzed by RRLC-MS/MS.

### Enzyme Kinetics Analysis

Kinetic parameters were assessed in the HLM, RLM, HKM, RKM, and recombinant human UGTs (UGT2B4, 2B7, and 1A9). Incubation conditions and sample preparation were as described in the Section “Glucuronidation Assay of NAF Enantiomers.” The concentrations of NAF-G were quantified by RRLC-MS/MS as described in the Section “Quantification of R(+)-NAF-G and S(-)-NAF-G.” Apparent *K*_m_ and *V*_max_ values were derived by fitting the triple experimental data to the appropriate model by non-linear least squares regression using GraphPad Prism 6.0 (GraphPad Software, San Diego, CA, United States). The models, which included the sample Michaelis–Menten model (Eq. 1), the substrate activation model (Eq. 2), and the substrate inhibition model (Eq. 3), were chosen based on the appearance of the Michaelis–Menten and Eadie–Hofstee plots.

(1) $$V={\text{V}}_{\max}[\text{S}]/K_{\text{m}}+[\text{S}]$$

(2) $$\text{V}={\text{V}}_{\max}{\left[\text{S}\right]}^{\text{N}}/K_{\text{m}}^{\text{N}}+S^{\text{N}}$$

(3) $$\text{V}={\text{V}}_{\max}[\text{S}]/K_{\text{m}}+[\text{S}](1+[\text{S}]/K_{\mathrm{si}})$$

where *V*_max_ is the maximal velocity, [S] is the substrate concentration, *K*_m_ is the Michaelis–Menten constant, N is an exponent indicative of the degree of curve sigmoidicity, and *K*_si_ is an inhibition constant. The apparent kinetic parameters were reported as mean ± standard error of the mean triplicate of samples.

### Inhibition of the Glucuronidation of NAF Enantiomers

Inhibition of the glucuronidation of NAF enantiomers was investigated using pooled HLM with the same incubation conditions as described in the section “Glucuronidation Assay of NAF Enantiomers.” As inhibitors for UGT2B4/UGT2B7 and UGT1A9, fluconazole (0.25 and 2.5 mM, final concentrations during incubation) and niflumic acid (5 and 50 μM) were added to the incubations. The concentrations of R(+)-NAF (50 μM) and S(-)-NAF (20 μM) corresponded to the approximate apparent *K*_m_ of glucuronidated NAF enantiomers of the pooled HLM.

### Inhibition of UGTs by R(+)-NAF and S(-)-NAF

The inhibition screening study was performed by investigating the 12 recombinant UGTs with 10 and 100 μM NAF enantiomer. TFP, which was used as the specific substrate of UGT1A4, was added to the incubated UGT1A4. 4-MU, as a non-specific substrate, was added to the other incubated samples. Instead of NAF enantiomers, the same volume of water was added to be the control sample. For different UGT incubation systems, incubation conditions were optimized according to the *K*_m_ value of 4-MU and TFP for individual recombinant UGTs (Supplementary Table 1). The formation of 4-MU-G and TFP-G produced from the 12 incubation system were analyzed by HPLC. The instrument parameters for the LC were as follows: system, Agilent 1260; Agilent Eclipse Plus C18 (2.1 mm × 50 mm, 1.8 μm, Santa Clara, CA, United States); mobile phase, methanol: 0.1% formic acid in water (0–3 min, 35:65; 3.1–4.5 min, 65:35; 4.6–5 min, 35:65); flow rate, 0.4 mL/min; column temperature, 25°C; and injection volume, 2 μL. The retention times of 4-MU, 4-MU-G, TFP, TFP-G, and MU (IS) were 1.5, 0.5, 2.2, 1.8, and 1.0 min, respectively.

The inhibition of UGT1A9 and UGT2B7 by NAF enantiomer was investigated using the pooled HLM, UGT1A9, and UGT2B7 with concentrations similar to that of the NAF enantiomer. Propofol, which was used as the specific substrate of UGT1A9, was added to the incubation. Zidovudine, as the specific substrate of UGT2B7, was added to the incubations (Supplementary Table 2 shows the incubation conditions). The formation of propofol glucuronide and zidovudine glucuronide was detected by RRLC-MS/MS. The condition was the same as that described in the Section “Quantification of R(+)-NAF-G and S(-)-NAF-G.” The following mass ion transitions (*m/z*) were used for detection: 353.3/177.2 for propofol glucuronide, 442.2/125.1 for zidovudine glucuronide, and 350.0/146.2 for meloxicam (IS). Inhibitor constants (*K*_i_ value) were estimated by fitting the expressions for competitive, non-competitive, and mixed inhibition models with Dixon plots and Lineweaver–Burk plots.

### Statistical Analysis

One-way ANOVA or Student’s *t*-test was used to analyze the data. The prior level of significance was set at *p* < 0.05.

## Results

### Quantification of R(+)-NAF-G and S(-)-NAF-G

**FIGURE 2:**
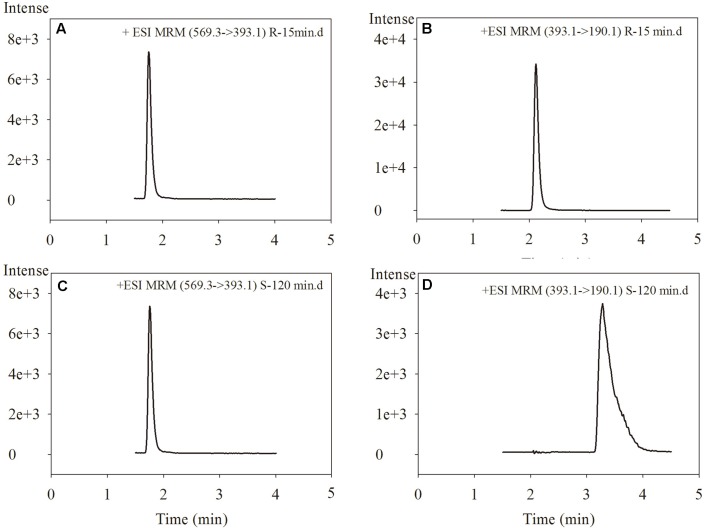
The chromatograms of R(+)-NAF-G **(A)**, R(+)-NAF **(B)**, S(–)-NAF-G **(C)**, and S(–)-NAF **(D)** in human liver microsome after incubation of R(+)-NAF for 15 min and of S(–)-NAF for 120 min.

Glucuronidations of NAF enantiomers were quantified as previously described (Liu et al., 2017). The retention times for R(+)-NAF-G, S(-)-NAF-G, NAF enantiomer, and IS were 1.8, 2.1, 3.2, and 2.9 min, respectively (**Figure 2**). No interfering signals were found at these retention times. The limit of detection for R(+)-NAF-G and S(-)-NAF-G was 0.8 nM. The standard curve were linear in the concentration range of 5 nM–100 μM for R(+)-NAF-G and S(-)-NAF-G in the microsomes and recombinant UGT enzymes. The equation used were as follows: *y* = 0.882*x* - 0.1285, *r* = 0.9997 for R(+)-NAF-G; and *y* = 0.8467*x* + 0.0108, *r* = 0.9998 for S(-)-NAF-G. The accuracy, inter-day, and intra-day precisions of R(+)-NAF-G and S(-)-NAF-G (2.5, 10, and 40 μM) were acceptable (data not shown).

### Glucuronidation of NAF Enantiomer in Rat and Human Tissue

**FIGURE 3:**
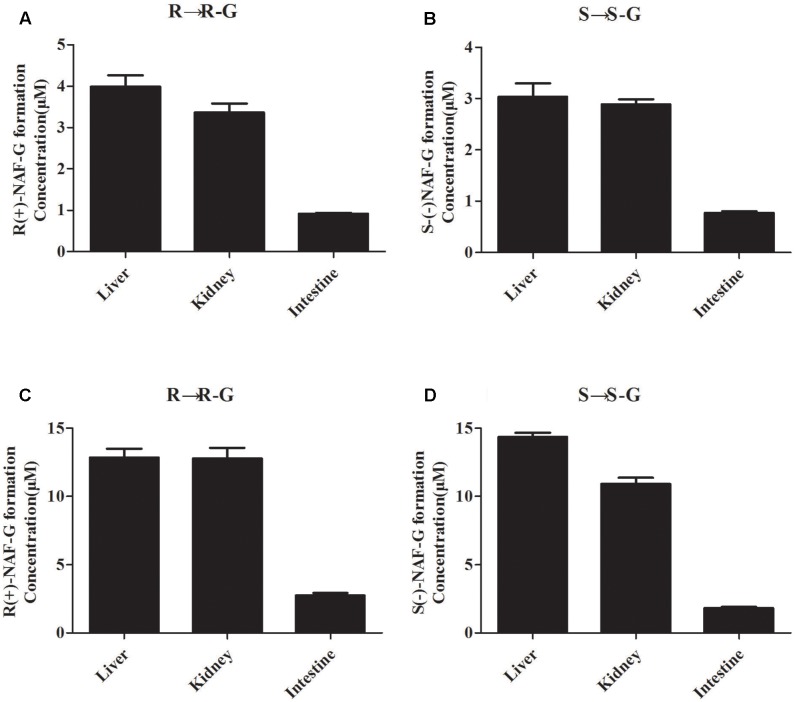
Glucuronide conjugation of naftopidil enantiomers by microsomes form rat and human liver, kidney, and intestine. Rat liver, kidney, and intestine microsomes were incubated with R(+)-NAF **(A)** and S(–)-NAF **(B)**, respectively. Human liver, kidney, and intestine microsomes were incubated with R(+)-NAF **(C)** and S(–)-NAF **(D)**, respectively. The formation of glucuronide were analyzed by LC-MS/MS. Data represent the mean ± standard error of triplicate samples. G, glucuronide.

In our previous study, we speculated that the glucuronidation of R(+)-NAF and S(-)-NAF occurred primarily in the liver of rat (Liu et al., 2017). Enantioselective metabolism of NAF in human has not been investigated. The liver, kidney, and intestine are considered the primary sites for drug glucuronidation *in vivo*. However, NAF glucuronidation in the kidney and intestine of rat and human has not been clarified. Enzymatic assays were using the HLM, HKM, HIM, RLM, RKM, and RIM. First, NAF glucuronides formed from the microsomes of rat and human tissues were the same as that detected in rat as described in our previous reports (Liu et al., 2017). In human tissue microsomes, the formation of NAF glucuronides were mainly produced by the liver and kidney, and much lesser produced by intestine (**Figures 3C,D**). Similar phenomenon was observed in rat tissue microsomes (**Figures 3A,B**). Furthermore, the amounts of glucuronides formed in the microsomes from human were more than that from rat (approximately five times).

### Glucuronidation of NAF Enantiomer by Recombinant Human Microsomes

**FIGURE 4:**
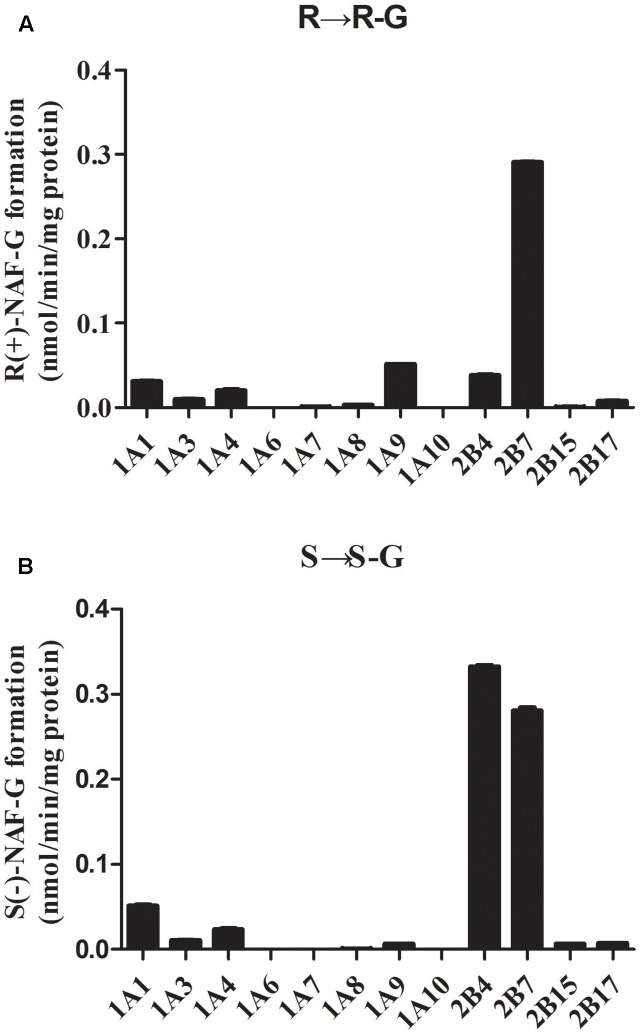
Screening of naftopidil enantiomers glucuronidation to form R(+)-NAF-G **(A)** and S(–)-NAF-G **(B)** with 12 recombinant human UGT enzymes (*n* = 3).

All available expressed UGTs were screened for NAF-UGT activity using 10 μM substrate concentration (**Figure 4**). UGT2B7 was the predominant isoform mediating R(+)-NAF glucuronidation with six times and nine times greater activity than UGT1A9 and UGT2B4, respectively. UGT1A1, 1A4, 1A3, 2B17, 1A8, 2B15, and 1A7 also mediated R(+)-NAF glucuronidation with more than 10 times lower activity than UGT2B7. No R(+)-NAF-G was detected with UGT1A6 and 1A10 (**Figure 4A**).

Interestingly, UGT2B4 showed relatively higher activity than UGT2B7 mediating S(-)-NAF-G. Both UGTs were the predominant isoforms with six to seven times higher activity than UGT1A1. UGT1A4, 1A3, 2B17, 2B15, 1A9, 1A8, and 1A7 also showed a small amount of activity. Similar to R(+)-NAF-G, UGT1A6 and 1A10 showed no activity for S(-)-NAF glucuronidation (**Figure 4B**).

### Kinetics of Glucuronidation of NAF Enantiomer by HLM, HKM, and Human Recombinant UGT Enzyme

Enzyme kinetic studies were performed using HLM, HKM, UGT2B7, 2B4, and 1A9. HLM and HKM showed similar glucuronidations of NAF enantiomer (**Figures 3C,D**). UGT2B7 exhibited higher activity than other human recombinant UGTs for NAF glucuronidation. Furthermore, differences were observed between the formation of R(+)-NAF-G and S(-)-NAF-G mediated by UGT2B4 and UGT1A9 (**Figure 4**). Therefore, we focused on the activities of these enzymes. Eadie–Hofstee plots were drawn to identify the kinetic model and estimate the kinetic parameters including affinity (*K*_m_), velocity (*V*_max_), and intrinsic clearance (*CL*_int_, the ratio *V*_max_/*K*_m_) (**Table 1**).

**Table 1:** Kinetic parameters of NAF glucuronidation by human tissue microsomes and recombinant UGT enzymes.

	*K*_**m**_ (μM)	*V*_**max**_ (pmol/min/mg)	*CL*_**int**_ (*V*_**max**_/*K*_**m**_) (μL/min/mg)	Kinetics type
R(+)-NAF-G
HLM	48 ± 3	3330 ± 131	70 ± 9	Michaelis–Menten
HKM	40 ± 4	2786 ± 138	70 ± 7	Michaelis–Menten
UGT2B7	21 ± 5	1043 ± 152	50 ± 3	Substrate inhibition
UGT2B4	51 ± 14	264 ± 38	5.2 ± 0.7	Substrate inhibition
UGT1A9	7 ± 1	34 ± 2	4.9 ± 1.1	Substrate inhibition
S(-)-NAF-G
HLM	19 ± 2	3472 ± 129	183 ± 17	Michaelis–Menten
HKM	35 ± 4	1957 ± 100	56 ± 10	Substrate inhibition
UGT2B7	38 ± 11	1331 ± 298	35 ± 6	Substrate inhibition
UGT2B4	55 ± 13	1976 ± 158	36 ± 3	Substrate inhibition
UGT1A9	3 ± 1	6.0 ± 2.2	2.3 ± 0.8	Michaelis–Menten

*Data are expressed as mean ± standard error of triplicate samples.*

**FIGURE 5:**
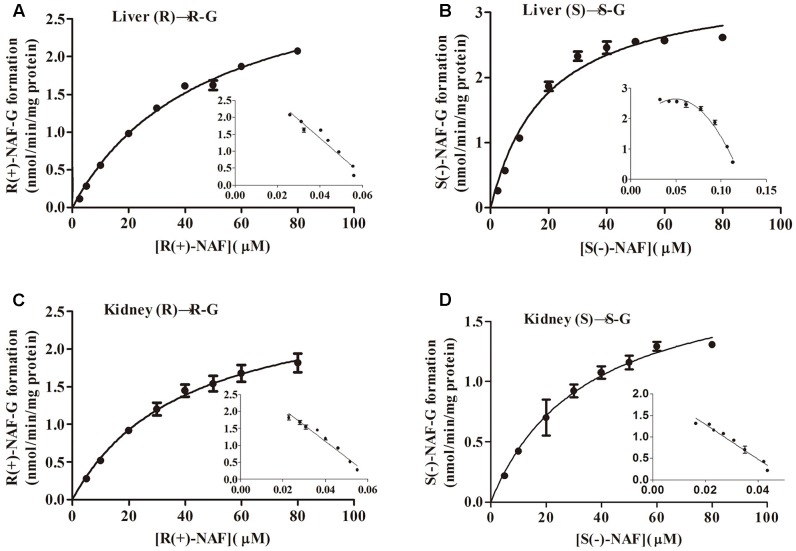
Kinetic analysis of naftopidil enantiomers conversion into their respective glucuronide conjugates by microsomes from human liver and kidney. Human liver microsomes were incubated with R(+)-NAF **(A)** and S(–)-NAF **(B)**. Human kidney microsomes were incubated with R(+)-NAF **(C)** and S(–)-NAF **(D)**. For each panel, large graphs represent the Michaelis–Menten plots, while small graphs correspond to the Eadie–Hofstee plots in triplicates.

Kinetic data for R(+)-NAF glucuronidation with HLM and HKM were best described by the Michaelis–Menten model, so was for S(-)-NAF glucuronidation with HLM, whereas the substrate inhibition model best described HKM for S(-)-NAF glucuronidation. The enzymatic affinities of HLM and HKM were higher for S(-)-NAF (19 μM for HLM and 35 μM for HKM) than that for R(+)-NAF (48 μM for HLM and 40 μM for HKM). Interestingly, the kinetics parameters for NAF enantiomers incubated with HKM were close. However, the activity for S(-)-NAF with HLM was more than two times greater than that for R(+)-NAF (more than two times lower in *K*_m_ value and two times higher in *CL*_int_ value) (**Table 1** and **Figure 5**).

**FIGURE 6:**
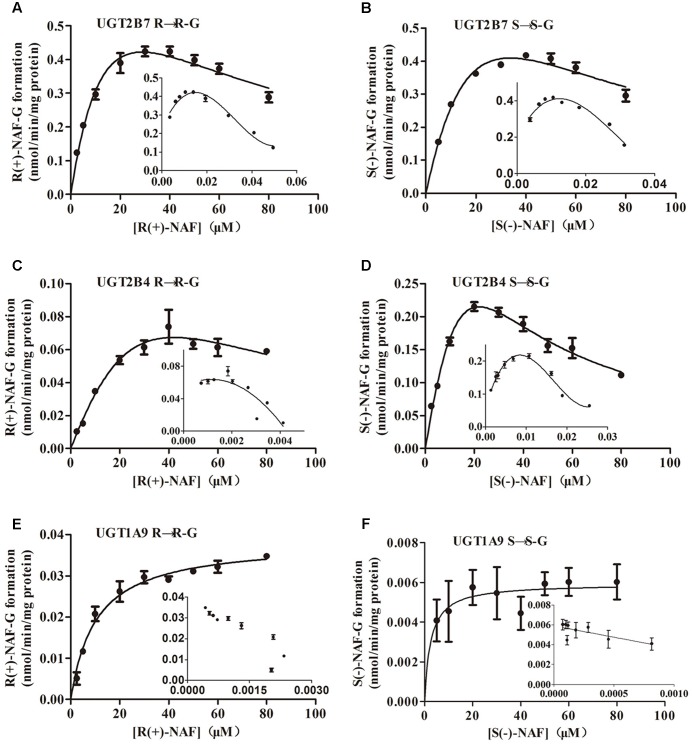
Kinetic analysis of naftopidil enantiomers conversion into their respective glucuronide conjugates by UGT1A9, UGT2B4, and UGT2B7. UGT2B7 was incubated with R(+)-NAF **(A)** and S(–)-NAF **(B)**. UGT2B4 was incubated with R(+)-NAF **(C)** and S(–)-NAF **(D)**. UGT1A9 was incubated with R(+)-NAF **(E)** and S(–)-NAF **(F)**. For each panel, large graphs represent the Michaelis–Menten plots, while small graphs correspond to the Eadie–Hofstee plots in triplicates.

Kinetic data for NAF glucuronidation with UGT2B7, 2B4, and 1A9 were best described by the substrate inhibition model, except the Michaelis–Menten model best described UGT1A9 for S(-)-NAF glucuronidation. *K*_m_ value for R(+)-NAF glucuronidation by HLM (48 μM) and HKM (40 μM) were most similar to that of UGT2B4 (51 μM). However, *CL*_int_ for UGT2B7 (50 μL/min/mg) was approximately 10 times higher compared with that of UGT2B4 (5.2 μL/min/mg). For S(-)-NAF glucuronidation, UGT2B4 showed similar kinetic parameters with UGT2B7 and HKM but significantly lower than HLM. UGT1A9 exhibited high affinity for both NAF enantiomers [*K*_m_ for R(+)-NAF 7 μM; *K*_m_ for S(-)-NAF 3 μM]. However, *V*_max_ and *CL*_int_ for both NAF enantiomers were much lesser than those of UGT2B4 and UGT2B7 (**Table 1** and **Figure 6**).

### Inhibition of Glucuronidation of NAF Enantiomer

**FIGURE 7:**
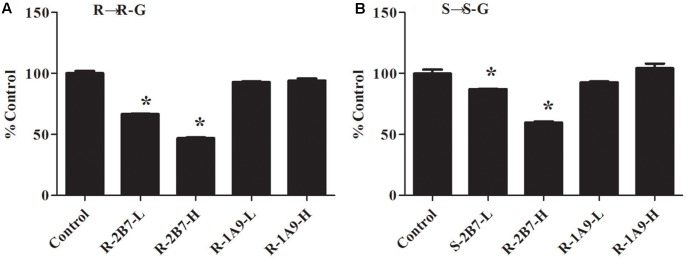
Inhibitory effect of specific inhibitor on the formation of R(+)-NAF-G **(A)** and S(–)-NAF-G **(B)** in human liver microsomes. Control represent R(+)-NAF-G and S(–)-NAF-G were incubated without inhibitor. While UGT2B-L and UGT2B-H groups represent NAF enantiomers incubated with fluconazole of 0.25 and 2.5 mM. UGT1A9-L and UGT1A9-H groups represent NAF enantiomers incubated with niflumic acid of 5 and 50 μM. The samples were processed and analyzed by LC-MS/MS as described in the Section “Quantification of R(+)-NAF-G and S(–)-NAF-G.” Each column represents mean ± standard error of triplicate samples. The asterisk “^∗^” indicates a statistically significant different (*p* < 0.05) to the control group, according to Student’s *t*-test.

The inhibition of NAF glucuronidation by specific inhibitors of UGT2B4/UGT2B7 and UGT1A9 is showed in **Figure 7**. Fluconazole, as inhibitor for UGT2B4 and UGT2B7, depressed the metabolism of NAF (0.25 mM inhibited approximately 35%; 2.5 mM inhibited approximately 55%). By contrast, niflumic acid (5, 50 μM), an inhibitor of UGT1A9, could not inhibit NAF glucuronidation.

### Inhibition of UGTs by R(+)-NAF and S(-)-NAF

**FIGURE 8:**
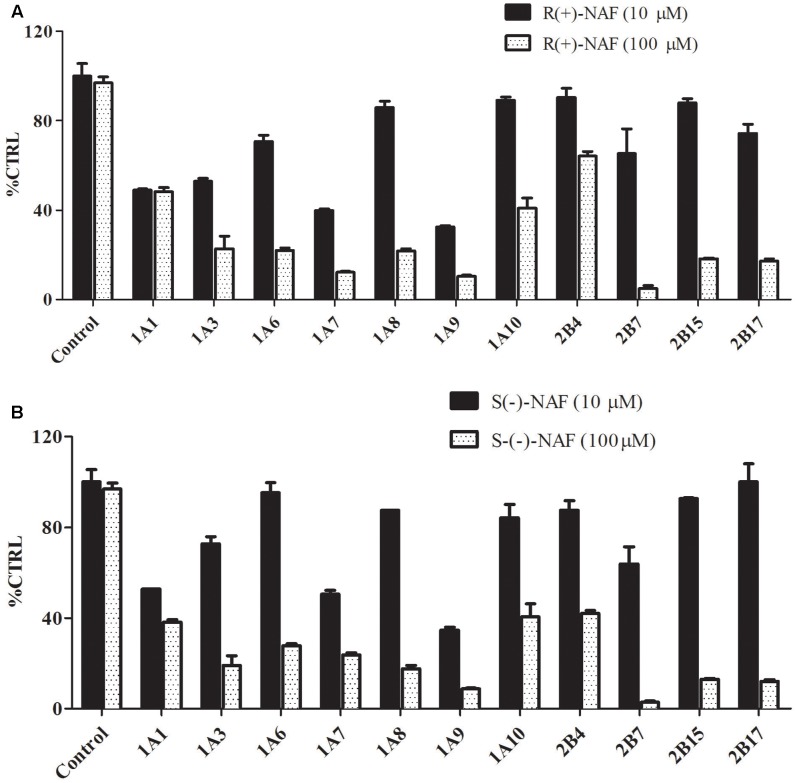
Inhibitory effect of R(+)-NAF **(A)** and S(–)-NAF **(B)** on the formation of TFP-G (typical probe substrates for UGT1A4) and 4-MU-G (unspecific probe substrate for other 11 UGT enzymes) in human liver microsomes in triplicate. Two concentration of R(+)-NAF and S(–)-NAF were added to the incubation. Control represent the formation of probe substrate glucuronide in human liver microsome without addition of R(+)-NAF and S(–)-NAF.

The inhibition screening results using the 12 recombinant UGTs by R(+)-NAF and S(-)-NAF are shown in **Figure 8**. The inhibition of 4-MU-G or TFP-G formation (above 50% by 10 μM NAF enantiomer and above 75% by 100 μM NAF enantiomer), was ordered as follows: for 10 μM NAF enantiomer, UGT1A9 > UGT2B7 > UGT1A1; and for 100 μM NAF enantiomer, UGT2B7 > UGT1A9 > UGT2B15 ≈ UGT2B17 ≈ UGT1A7. No significant difference was detected between R(+)-NAF and S(-)-NAF. This result indicated that UGT2B7 not only played the predominant role for NAF glucuronidation, but was also inhibited by NAF enantiomers. Although the contribution of UGT1A9 for NAF glucuronidation was small, UGT1A9 could be significantly inhibited by R(+)-NAF and S(-)-NAF even at low concentrations.

**FIGURE 9:**
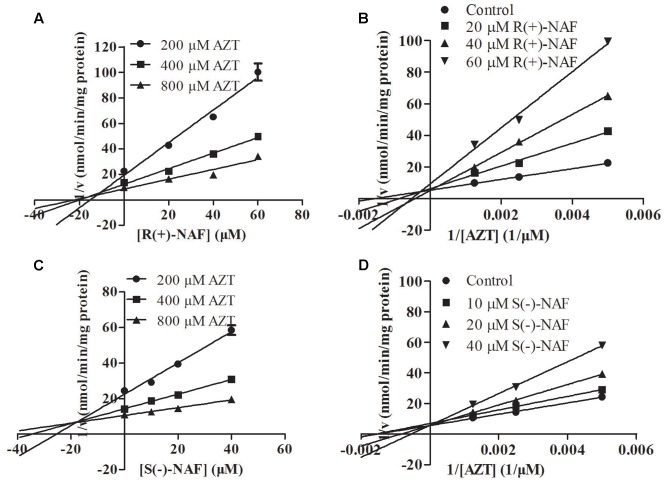
Representative Dixon plots **(A,C)** and Lineweaver–Burk plots **(B,D)** of the effect of R(+)-NAF and S(–)-NAF on AZT glucuronide formation in recombinant UGT2B7. Data points shown represent the mean ± standard error of triplicate samples.

**FIGURE 10:**
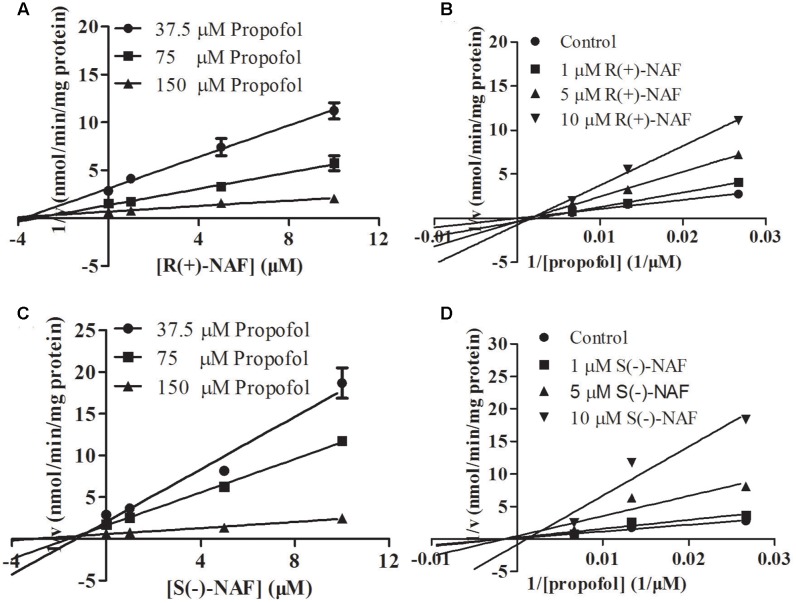
Representative Dixon plots **(A,C)** and Lineweaver–Burk plots **(B,D)** of the effect of R(+)-NAF and S(–)-NAF on propofol glucuronide formation in recombinant UGT1A9. Data points shown represent the mean ± standard error of triplicate samples.

We also investigated the inhibition of UGT2B7 and UGT1A9 using their specific substrates with pooled HLM, UGT1A9, and UGT2B7 (**Table 2**). We identified the inhibition type and estimated *K*_i_ using the Dixon and Lineweaver–Burk plots (**Figures 9**, **10**). The NAF enantiomers inhibition of UGT1A9 was best described by the non-competitive model, while the inhibition of UGT2B7 by these enantiomers was best fitted by the competitive model. In HLM and recombinant UGTs, R(+)-NAF and S(-)-NAF showed significantly stronger inhibition to UGT1A9 with much lower *K*_i_ value than that to UGT2B7 (**Table 2** and Supplementary Figures 1–4).

**Table 2:** The inhibition kinetic type and parameters (*K*_i_) of UGT1A9 and UGT2B7 by R(+)-NAF and S(-)-NAF.

Substrate	Enzyme	Inhibitor			
		R(+)-NAF		S(-)-NAF	
		Inhibition type	*K*_i_ (μM)	Inhibition type	*K*_i_ (μM)
Propofol	HLM	Non-competitive	10.0 ± 1.9	Non-competitive	11.5 ± 2.0
Zidovudine	HLM	Competitive	64.9 ± 7.8	Competitive	35.7 ± 4.1
Propofol	UGT1A9	Non-competitive	3.6 ± 0.7	Non-competitive	1.0 ± 0.2
Zidovudine	UGT2B7	Competitive	12.1 ± 2.2	Competitive	18.5 ± 3.3

*Data are expressed as mean ± standard error of triplicate samples.*

## Discussion

Previous studies identified three phase I metabolites and two phase II conjugations in rat plasma containing NAF glucuronides and hydroxyl-NAF glucuronides (Niebch et al., 1991; Li et al., 2006). Three phase I metabolites, NHN, PHN, and DMN, required catalytic activity by CYP2C9 and CYP2C19 (Zhu et al., 2014). It was not possible to quantify levels of these metabolites, so contributions of CYP450s and UGTs to the metabolism of NAF remain unclear. Our previous study compared metabolites and parent drugs in rat plasma. Our findings showed that levels of R(+)-NAF-G and S(-)-NAF-G were much higher than those of other metabolites, including R(+)-NAF and S(-)-NAF, NHN, PHN, and DMN (Liu et al., 2017). Thus, we confirmed that glucuronidation plays a primary role in the biotransformation of NAF. In first-pass metabolism of NAF, the contribution of UGTs was superior to that of CYP450s.

We previously developed and validated an RRLC-MS/MS method to quantify levels of NAF enantiomer glucuronides in rat (Liu et al., 2017). This method was employed in the present study, with partly validation, including matrix effect, selectivity, calibration curve, accuracy, and precision. In this study, the major metabolites formed from human and rat tissue microsomes exhibited the same chromatographic and MS performance as previously reported. We inferred that the glucuronides of NAF formed *in vitro* were similar in nature to those formed *in vivo*. Investigations of NAF metabolism *in vitro* should therefore inform our discussions of metabolism in humans.

Our data indicated that, whether in human or rat, liver and kidney exhibited similar contributions to the clearance of NAF enantiomers *in vivo*. Thus, we suspected that the metabolism of NAF was similar in human and rat. The enzymes involved in NAF metabolism in rat and human exhibit substantial homology. These enzymes are distributed primarily in liver and kidney. Intestine did not clear substantial amounts of NAF enantiomer. The bioavailability of NAF was low in both human (<20%) and rat (6.8–14.5%) (Himmel, 1994; Liu et al., 2015, 2017). Our study revealed that hepatic, urinary, and intestinal glucuronidation metabolisms are all closely associated with low oral bioavailability. Metabolisms of human liver and kidney glucuronidation for R(+)-NAF were similar, with close *K*_m_, *V*_max_, and *CL*_int_ values. However, for S(-)-NAF, liver glucuronidation metabolism was approximately threefold greater than kidney glucuronidation metabolism, with a *K*_m_ value that was threefold lower and a *CL*_int_ value that was threefold greater. Compared with liver glucuronidation metabolism of R(+)-NAF and S(-)-NAF, S(-)-NAF showed decreased *K*_m_ and increased *V*_max_ and *CL*_int_. This observation was in agreement with the quicker metabolism rate of S(-)-NAF, as observed in rats receiving oral doses of NAF enantiomers (Liu et al., 2015).

Screening for the reactivity of UGT-catalyzed R(+)-NAF and S(-)-NAF revealed the elevated capacities of UGT2B7, 2B4, and UGT1A9 to convert NAF enantiomers to their glucuronides. Various findings revealed UGT2B7 to be a dominant player in these reactions. First, UGT2B7 exhibited high reactivity for both R(+)-NAF and S(-)-NAF, while UGT2B4 showed elevated capacity only for S(-)-NAF, and UGT1A9 showed relatively elevated capacity only for R(+)-NAF. For both NAF enantiomers, UGT2B7, rather than UGT1A9, exhibited the most similar *V*_max_ and *CL*_int_ values to the liver or kidney microsome. UGT2B4 showed considerably lower activity than UGT2B7 in mediating the glucuronidation of R(+)-NAF. However, when mediating the metabolism of S(-)-NAF, UGT2B4 and UGT2B7 played major roles, with similar kinetics. UGT2B7 was quantified by LC-MS/MS as the most abundant UGT isoform in liver and kidney, decreased levels were observed in intestine (Fallon et al., 2013; Sato et al., 2014). Thus, lower glucuronidation activity was detected in intestine with microsomal proteins. Because of the lack of a widely accepted specific inhibitor for UGT2B4 and UGT2B7, we used fluconazole to inhibit UGT2B4 and UGT2B7 (Oda et al., 2015). The results indicated that UGT2B4 and UGT2B7 were closely associated with this reaction, while UGT1A9 barely participated.

Differences between the metabolism of R(+)-NAF and S(-)-NAF *in vivo* and *in vitro* were caused by the selective catalysis of UGT2B4 during glucuronidation of S(-)-NAF. Analysis of mRNA levels and quantification by LC-MS/MS showed UGT2B4 to be abundant in liver, but absent in small intestine (Ohno and Nakajin, 2008; Fallon et al., 2013; Sato et al., 2014). However, UGT2B4 primarily catalyzed the metabolism of S(-)-NAF and only minimally catalyzed glucuronidation of R(+)-NAF. No difference in enantiomer glucuronidation was detected in HIM, but HLM catalyzed glucuronidation of S(-)-NAF by UGT2B4 with much higher efficiency. This result was also consistent with the previous demonstration that S(-)-NAF-G in plasma peaked more quickly than did levels of R(+)-NAF-G (Liu et al., 2015).

Kinetic parameters for UGT1A9 included dramatically decreased *K*_m_ value for R(+)-NAF (7 ± 1 μM) and S(-)-NAF (3 ± 1 μM), compared with kinetics of UGT2B7 [21 ± 5 μM for R(+)-NAF and 38 ± 11 μM S(-)-NAF]. These findings indicate that UGT1A9 had higher affinity than UGT2B7 for R(+)-NAF and S(-)-NAF. However, the markedly lower *V*_max_ and *CL*_int_ values of UGT1A9, compared with those of UGT2B7 and UGT2B4, suggest that the reaction capacity of UGT1A9 was lower than that of UGT2B7 and UGT2B4. We suspect that R(+)-NAF and S(-)-NAF may have inhibited the activity of UGT1A9. We also evaluated the potential inhibition of UGT isoforms by R(+)-NAF and S(-)-NAF. Our data revealed that R(+)-NAF and S(-)-NAF non-competitively inhibited UGT1A9 at low concentration (<10 μM) and competitively inhibited UGT2B7 at relatively higher concentration (10–100 μM). After oral treatment, human plasma levels of NAF are typically below 10 μM, which suggests that clinical doses of NAF may interfere with UGT1A9 function. NAF was administered clinically to elderly male patients, who received a combination of various classes of drugs, all of which are eliminated by UGT1A9. UGT1A9 is the predominant enzyme catalyzing glucuronidation of edaravone, furosemide, and phenylbutazone (Sehrt et al., 2005; Nishiyama et al., 2006; Ma et al., 2012). Patients taking these drugs in combination with NAF carry high risk for drug–drug interaction.

UGT1A9 also plays a significant role in modulating the availability of arachidonic acid for biosynthetic pathways and providing a local intra-renal detoxification pathway *in vivo* (Knights et al., 2013; Miners et al., 2017). Various investigations have shown that UGT1A9 is a polymorphic enzyme (Yamanaka et al., 2004; Kuypers et al., 2005). Effects on the activity of UGT1A9 may result in more complex drug–drug interactions and drug–endobiotic interactions. Our study highlighted a potential risk for drug–drug interactions when combining UGT1A9 substrates.

## Conclusion

This study was the first to report on individual glucuronidation of R(+)-NAF and S(-)-NAF *in vitro* in rat and human. For these NAF enantiomers, hepatic and kidney glucuronidation were the major metabolic pathways. UGT2B7 was identified as the principal enzyme mediating the glucuronidation of R(+)-NAF and S(-)-NAF. UGT2B4 played a key role in the stereoselective metabolism of NAF enantiomers. R(+)-NAF and S(-)-NAF also showed potential inhibition of UGT1A9.

## Author Contributions

X-WL and C-FL conceived and designed the experiments. YR and X-FZ performed the experiments. X-WL and YR analyzed the data. J-JH, YC, B-YH, LZ, BW, and NH contributed the reagents/materials/analysis tools. X-WL and C-FL wrote the paper.

## Conflict of Interest Statement

The authors declare that the research was conducted in the absence of any commercial or financial relationships that could be construed as a potential conflict of interest.
