# Supplementary material for: Human UDP-Glucuronosyltransferase 2B4 and 2B7 Are Responsible for Naftopidil Glucuronidation in Vitro
Source: Front Pharmacol. 2018 Jan 11;8:984. doi: 10.3389/fphar.2017.00984 (PMC5769128; doi:10.3389/fphar.2017.00984)
Supplement: Supplementary file 1 [file Presentation_1.PDF]

Supplementary Material

Enantiospecific glucuronidation of naftopidil by human in vitro: UDP-glucuronosyltransferase 2B4 and 2B7 are the principal responsible enzymes

Xia-Wen Liu[*], Yi Rong, Xing-Fei Zhang, Jun-Jun Huang, Yi Cai, Bi-Yun Huang, Liu Zhu, Bo Wu, Ning Hou, Cheng-Feng Luo*

*** Correspondence:** Xia-Wen Liu melody_12@163.com

Cheng-Feng Luo rocenphone@hotmail.com

1 Supplementary Figures and Tables

1.1 Supplementary Tables

Supplementary Table1. Incubation conditions for the inhibition screening by NAF enantiomers.

UGTs	Protein (mg/mL)	Substrate (μM)	Incubation time (min)
UGT1A1	0.1	100	120
UGT1A3	0.05	1000	75
UGT1A4	0.1	40	20
UGT1A6	0.05	100	30
UGT1A7	0.05	15	30
UGT1A8	0.05	750	30
UGT1A9	0.025	10	30

UGT1A10	0.1	30	120
UGT2B4	0.5	1000	120
UGT2B7	0.025	300	120
UGT2B15	0.2	250	120
UGT2B17	0.2	2000	120

Supplementary Table2. Incubation conditions for the inhibition dynamics study by NAF enantiomers

Enzyme	Protein (mg/mL)	Substrate	Substrate concentration (μM)	Inhibitor concentration (μM)	Time (min)
HLM	0.1	Propofol	50, 100, 200	0, 5, 10, 25	30
UGT1A9	0.05	Propofol	37.5, 75, 150	0, 1, 5, 10	30
HLM	0.1	AZT	250, 500, 1000	0, 25, 50, 100	30
UGT2B7	0.1	AZT	200, 400, 800	0, 20, 40, 60 /0, 10, 20, 40	30

1.2 Supplementary Figures

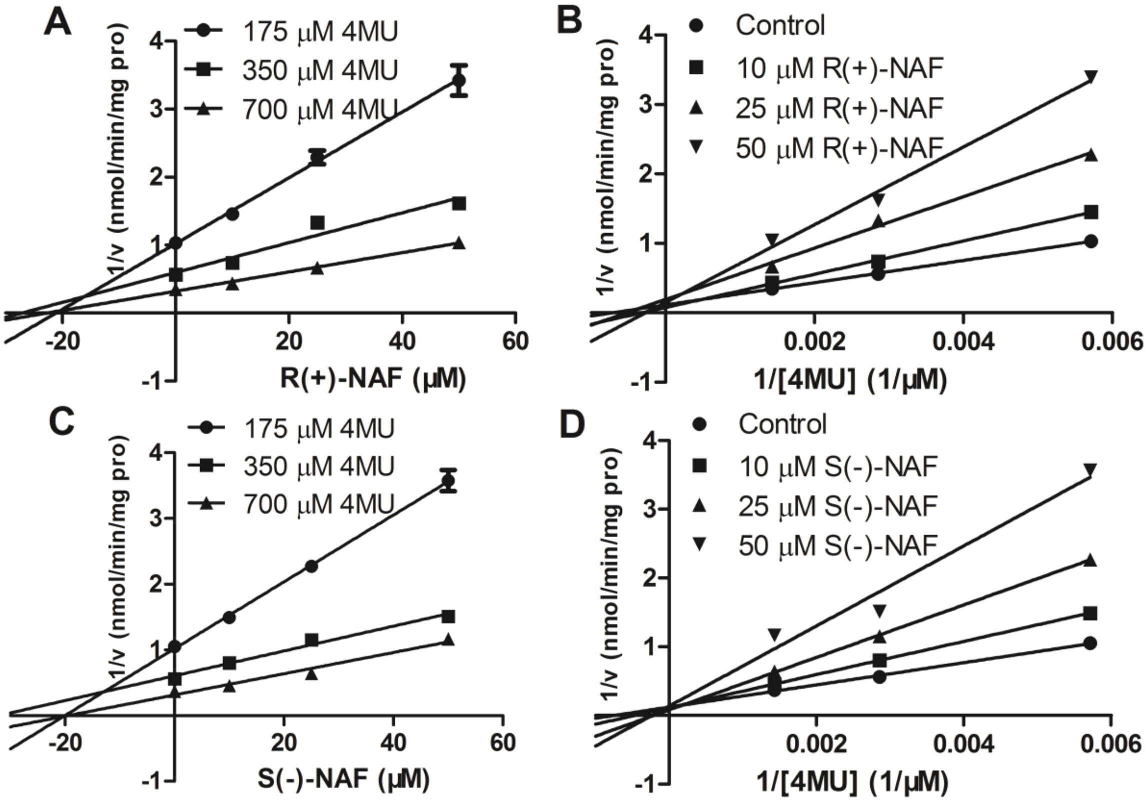

Supplementary Figure 1. Representative Dixon plots (A and C) and Lineweaver-Burk plots (B and D) of the effect of R(+)-NAF and S(-)-NAF on 4-MU glucuronide formation in recombinant UGT2B7. Data points show represent the mean ± standard error of triplicate samples.

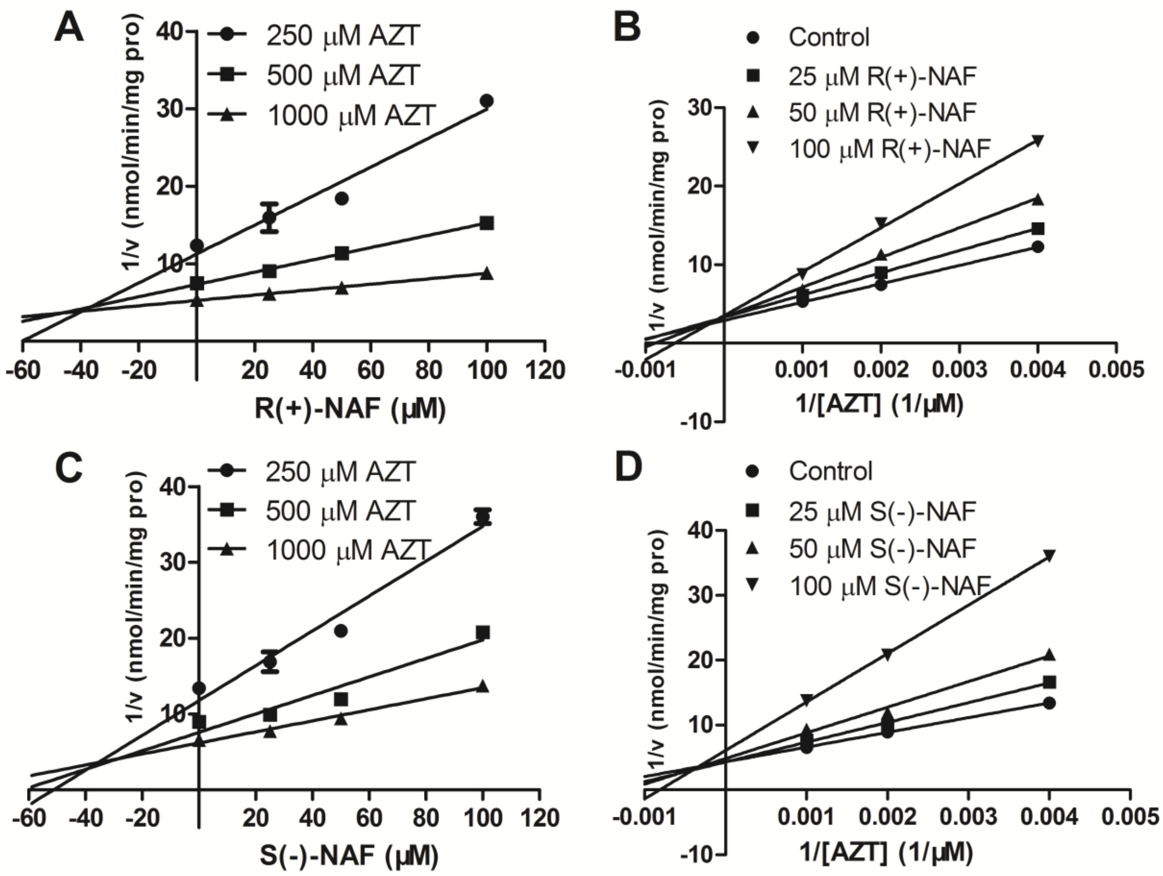

Supplementary Figure 2. Representative Dixon plots (A and C) and Lineweaver-Burk plots (B and D) of the effect of R(+)-NAF and S(-)-NAF on AZT glucuronide formation in pooled human liver microsomes. Data points show represent the mean ± standard error of triplicate sample

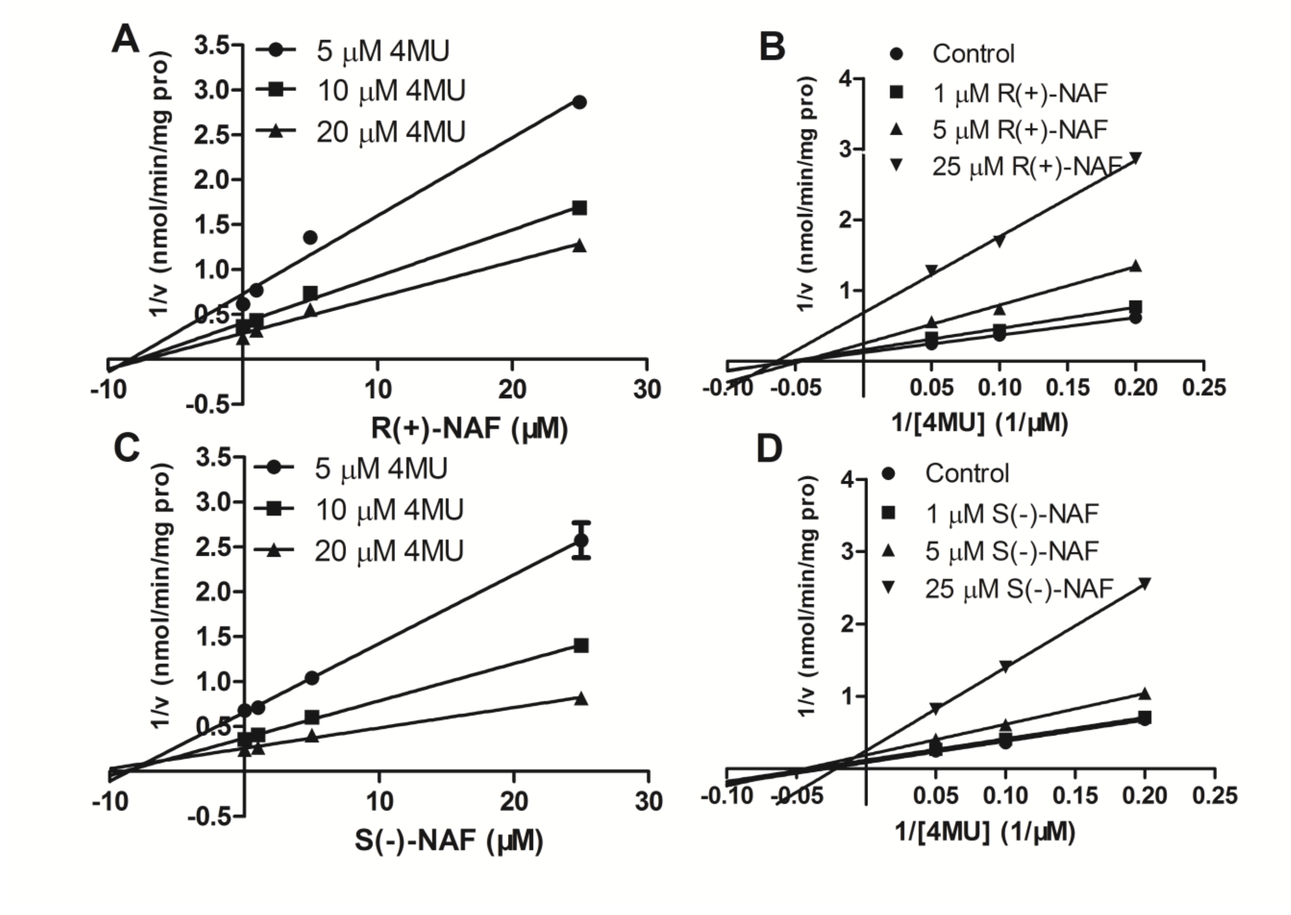

Supplementary Figure 3. Representative Dixon plots (A and C) and Lineweaver-Burk plots (B and D) of the effect of R(+)-NAF and S(-)-NAF on 4-MU glucuronide formation in recombinant UGT1A9. Data points show represent the mean ± standard error of triplicate samples.

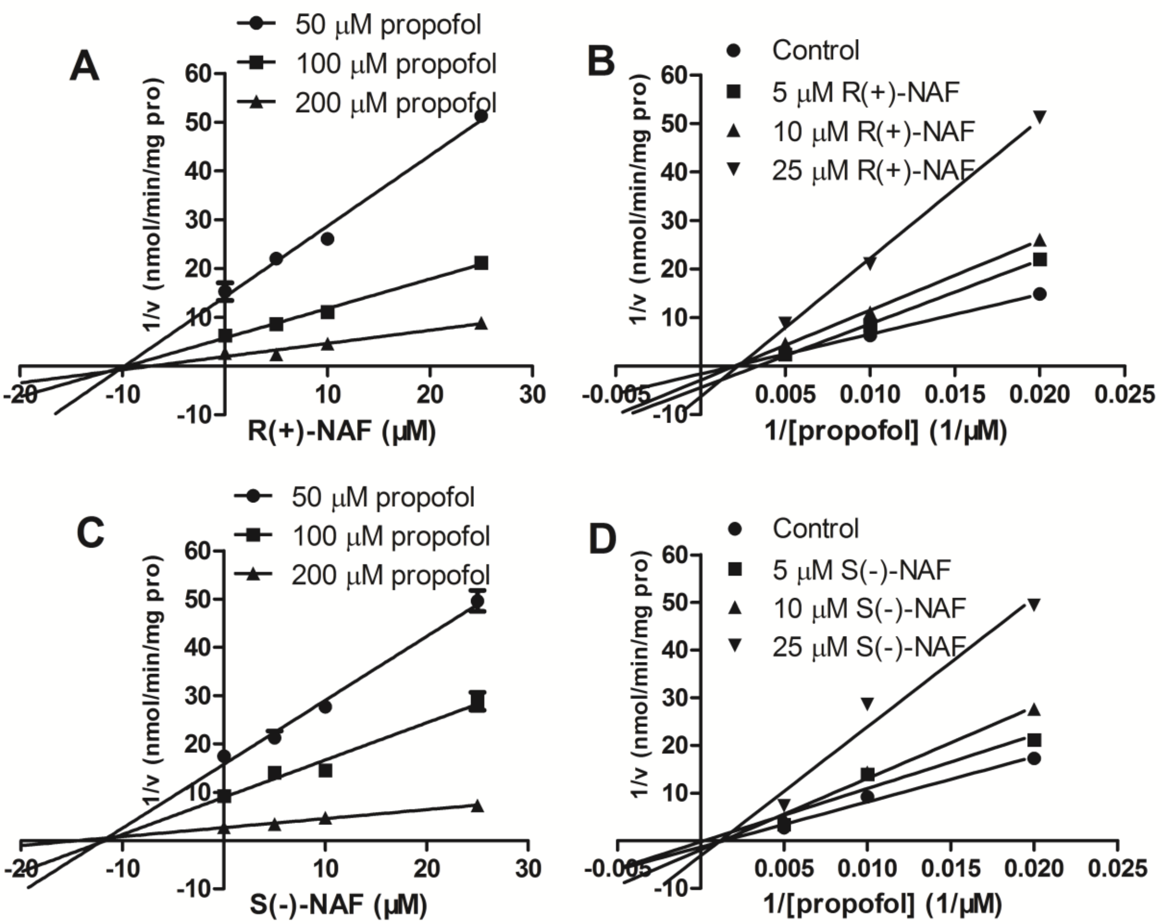

Supplementary Figure 4. Representative Dixon plots (A and C) and Lineweaver-Burk plots (B and D) of the effect of R(+)-NAF and S(-)-NAF on propofol glucuronide formation in pooled human liver microsomes. Data points show represent the mean ± standard error of triplicate samples.
